# Three-dimensional imaging of the extracellular matrix and cell interactions in the developing prenatal mouse cornea

**DOI:** 10.1038/s41598-019-47653-z

**Published:** 2019-08-02

**Authors:** Eleanor M. Feneck, Philip N. Lewis, Keith M. Meek

**Affiliations:** Structural Biophysics Research Group, School of Optometry and Vision Sciences, Cardiff University, Maindy Road, Cardiff, CF24 4HQ UK

**Keywords:** Electron microscopy, Embryology

## Abstract

As the outer lens in the eye, the cornea needs to be strong and transparent. These properties are governed by the arrangement of the constituent collagen fibrils, but the mechanisms of how this develops in mammals is unknown. Using novel 3-dimensional scanning and conventional transmission electron microscopy, we investigated the developing mouse cornea, focusing on the invading cells, the extracellular matrix and the collagen types deposited at different stages. Unlike the well-studied chick, the mouse cornea had no acellular primary stroma. Collagen fibrils initially deposited at E13 from the presumptive corneal stromal cells, become organised into fibril bundles orthogonally arranged between cells. Extensive cell projections branched to adjacent stromal cells and interacted with the basal lamina and collagen fibrils. Types I, II and V collagen were expressed from E12 posterior to the surface ectoderm, and became widespread from E14. Type IX collagen localised to the corneal epithelium at E14. Type VII collagen, the main constituent of anchoring filaments, was localised posterior to the basal lamina. We conclude that the cells that develop the mouse cornea do not require a primary stroma for cell migration. The cells have an elaborate communication system which we hypothesise helps cells to align collagen fibrils.

## Introduction

The cornea’s biomechanical strength and optical transparency are governed by the ability of collagen fibrils to assemble into organised lamellae, under the influence of proteoglycans controlling collagen fibril diameter and biosynthesis^1,2^. Extensive research has been carried out to understand the developing corneal structure within the avian cornea, but knowledge of the composition, distribution and organisation of extracellular matrix components within the developing mammalian cornea is woefully lacking, and this is important as there are structural differences between the mature chick cornea and the mature mammalian cornea^3,4^. Analysing the structural properties of the mammalian cornea during its initial development is important to elucidate the mechanisms underlying mature tissue function, and its failure in corneal developmental abnormalities.

The initial development of the avian cornea is seen with the surface ectoderm secreting an acellular primary stroma composed of types I, II, V and IX collagen^5,6^. Type IX collagen breakdown activates the swelling of the primary stroma, initiating the migration of mesenchymal cells^7,8^. These cells proceed to synthesise the secondary corneal stroma, which eventually becomes the mature corneal stroma. Types II and IX collagen are seen to form heterotypic fibrils within the primary stroma. Once mesenchymal invasion is complete, type IX collagen is undetectable but type II collagen increases^9^. After approximately day 10 of avian development, type II collagen is synthesised from the mesenchymal cells, replacing the synthesis of type I collagen^10^. As the secondary stroma matures, the most prevalent collagen fibril types are type I and V collagen, which form heterotypic fibrils that maintain collagen fibril diameter^11,12^. The identification of the collagen types and extracellular matrix interactions within avian development has led to a greater understanding of the developmental events and the components required to achieve avian corneal transparency. The mammalian cornea is already considered to have key developmental differences compared to the avian cornea. Within mammalian development, the lack of secretory organelles within the corneal epithelium alongside the unidentifiable organised acellular matrix layer has led to the proposition that the mammalian cornea does not require a primary stroma^13^. The proposed absence of the primary stroma suggests that different mechanisms and events occur in the developing mammalian cornea.

The secretion and alignment of collagen fibrils within the extracellular matrix of the developing mammalian cornea is also poorly understood. Studies that have analysed collagen fibril assembly within prenatal tendon development have identified collagen being transported from the Golgi apparatus into fibripositors that deposit and align collagen fibrils^14–17^. This theory of collagen fibril deposition has also been suggested to occur during avian corneal development^16^, but has not been seen in the mammalian cornea.

Further studies have identified that keratocytes within the avian cornea associate with collagen fibril organisation^16^. It has also been shown that corneal stromal cells rotate, with the subsequent alignment of collagen fibrils forming successively rotating lamellae^18^. However, the underlying mechanisms regulating collagen assembly and the organisation of collagen lamellae into an orthogonal arrangement is unknown. Elucidating the mechanisms underlying the somewhat different collagen arrangement in the mammalian cornea will lead to a greater understanding of how the mammalian cornea achieves transparency through development, and why there seem to be similarities, but some fundamental differences, between the avian and mammalian cornea.

This paper aims to investigate, using 3-D imaging techniques, the development of the prenatal mouse cornea, to determine if a primary stroma is present, and to elucidate the cellular mechanisms that direct collagen lamellar formation. To our knowledge, this paper is the first to provide a comprehensive 3-D electron microscopy study of the developing mouse cornea using serial block face scanning electron microscopy (SBF-SEM).

## Results

### General ultrastructural morphology

#### Stage E10

**Figure 1:**
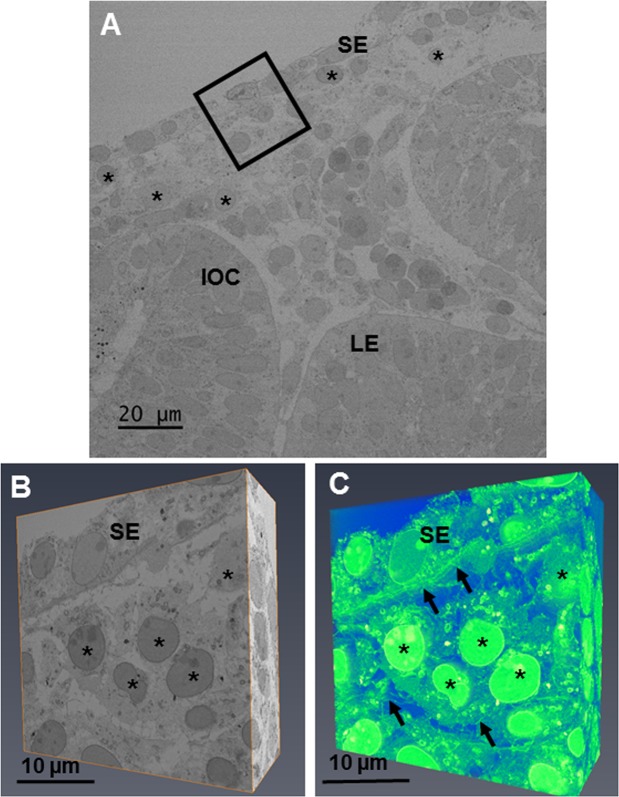
The developing eye at E10 was imaged at a low magnification (×1.19 K) to show the lens epithelium (LE), inner layer of the optic cup (IOC) and surface ectoderm (SE). (**A**) Once orientated, an area of the developing cornea (black box) was chosen for analysis at high magnification (×4.33) with serial-block face scanning electron microscopy (SBF-SEM). (**A**) The chosen area was imaged between the surface ectoderm and the migrating neural crest cells on the SBF-SEM. (**B**) 3-D models of the dataset were made by selecting the volren function in Amira, which automatically segmented the cells and extracellular matrix. (**C**) The reconstructions showed neural crest cells which had migrated into the presumptive corneal stroma. No collagen fibrils were seen within this region of the cornea. The cells were round (black asterisks) with cell projections reaching to adjacent neural crest cells. Extracellular strands were also seen to run through the basal lamina, communicating with cells of the surface ectoderm and the neural crest cells (black arrows). (**C**) To see the reconstructions in more detail and a clearer view of the cell extensions, please refer to Supplementary Video 1.

**Figure 2:**
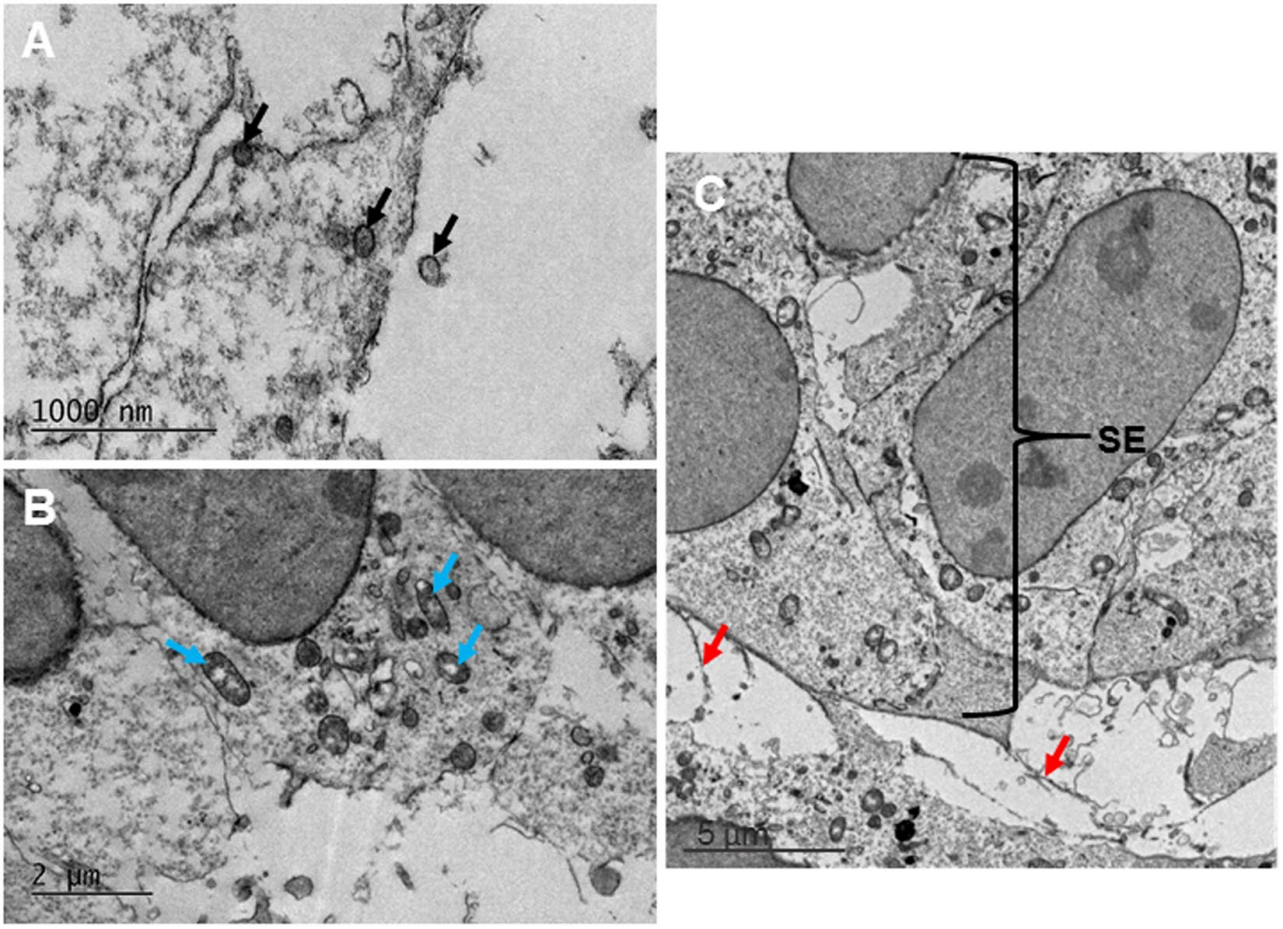
Transmission electron microscopy imaging showed high resolution images of the developing cornea at E10. High resolution images of the neural crest cells that had migrated into the area of the developing cornea showed a large quantity of organelles and vesicles intracellularly and extracellularly (black arrows). (**A**) Synthesising organelles were also seen within the mesenchymal cells, including mitochondria, endoplasmic reticulum and Golgi apparatus (blue arrows). (**B**) Extracellular material (red arrows) extended from the cells of the surface ectoderm (SE) into the developing corneal stroma (**C**).

At developmental age E10, the eye was seen to have a surface ectoderm overlying the developing lens epithelium and inner layer of the optic cup (Fig. 1). Neural crest cells with a round morphology populated the area between the lens and surface ectoderm (Fig. 1). The 3-D reconstructions of the eye at E10 showed that these neural crest cells possess extensions spanning to neighbouring cells. Thin strands were shown to penetrate through the surface ectoderm, as well as interacting with the neural crest cells (Fig. 1 and Supplementary Video 1). High resolution images using transmission electron microscopy showed no sign of an organised acellular collagen matrix layer (Fig. 2). The cells within the area of the presumptive corneal stroma contained small intracellular and extracellular vesicles. In addition, synthesising organelles, including endoplasmic reticulum and Golgi apparatus, were seen within the corneal stromal cells. The cells of the surface ectoderm were also seen to have extracellular matrix extensions whose nature is unknown (Fig. 2). No acellular organised collagen matrix was seen within the developing cornea at E10. Measurement of the number of corneal cells in the corneal stroma indicated ~3 cells within a 1000 μm^3^ volume.

#### Stage E12

**Figure 3:**
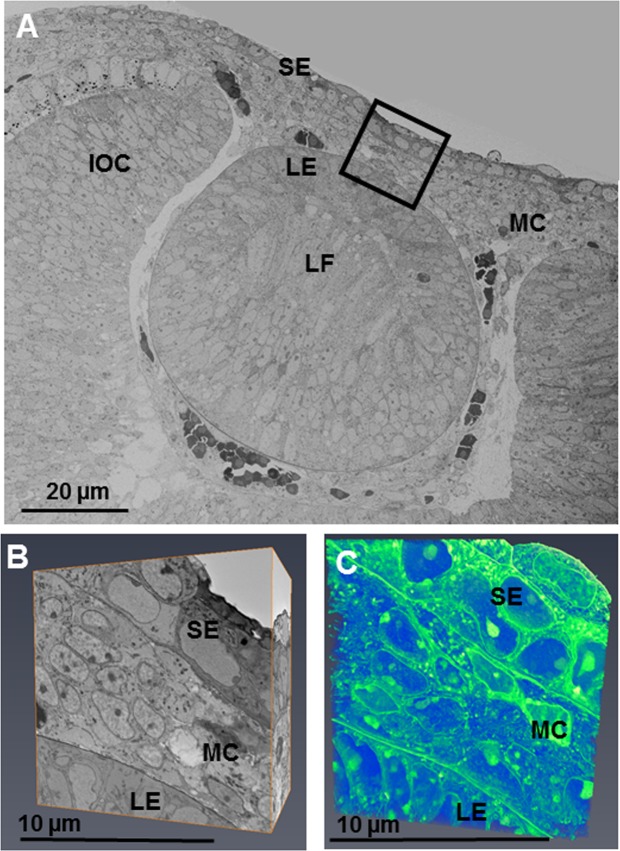
The developing eye at E12 was imaged at a low magnification (×724 K) and images stitched together to show the developing lens epithelium (LE), lens fibres (LF), surface ectoderm (SE), mesenchymal cells (MC) and inner layer of the optic cup (IOC). (**A**) Reconstructions within the area of the black box were made following the method described in Fig. 1. (**A**,**B**) The reconstructions showed all cells in the developing corneal stroma to be in close proximity to one another, appearing to indicate a condensation of cells. (**B**,**C**) To see the cell reconstructions in more detail, please refer to Supplementary Video 2.

**Figure 4:**
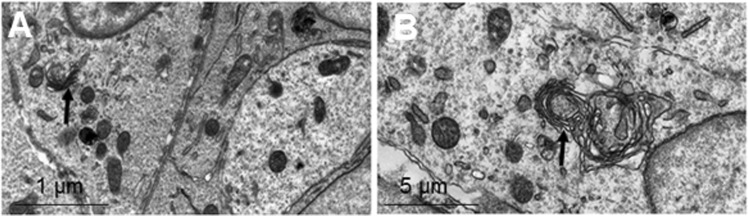
Transmission electron microscopy showed high resolution images of the developing cornea at E12. (**A**,**B**) The mesenchymal cells located between the surface ectoderm and the lens were in very close proximity to neighbouring cells. The cells contained mitochondria and synthesising organelles including endoplasmic reticulum and Golgi apparatus (black arrows).

At embryonic day E12, the mesenchymal cells between the lens and surface ectoderm had increased in density compared to E10, with quantitative analysis showing ~12 corneal stromal cells in a 1000 μm^3^ volume compared with ~3 cells at E10. All cells in the presumptive stroma were in close proximity to one another (Fig. 3). The supplementary video accompanying Fig. 3 shows mesenchymal cells with a stellate morphology and a denser cytoplasm. This indicates that some cells have a more mature phenotype, which suggests a change in cell type. High resolution imaging revealed that the mesenchymal cells contained many synthesising organelles such as endoplasmic reticulum and Golgi apparatus (Fig. 4).

#### Stage E13

**Figure 5:**
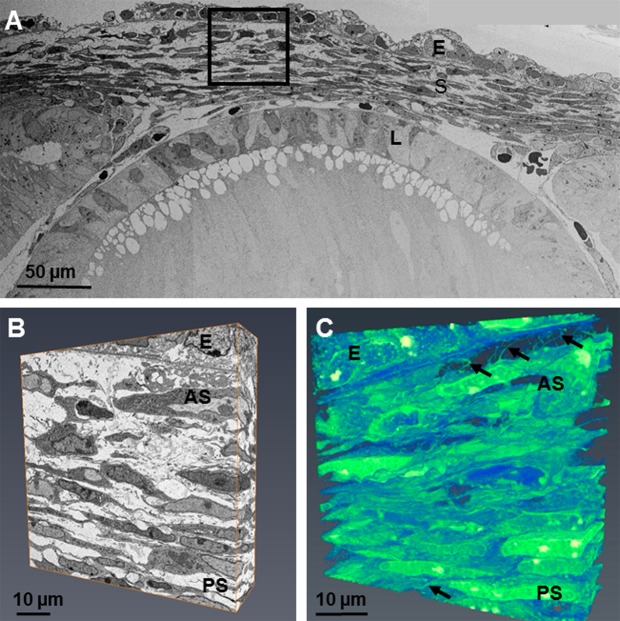
The developing eye at E13 was imaged at a low magnification (×724) and images stitched together to show the lens (L), corneal stroma (S) and corneal epithelium (E). (**A**) Reconstructions at a high magnification within the area of the black box were made following the method described in Fig. 1. (**B**) The reconstructions showed that the cell morphology within the anterior stroma was stellate whereas the cells in the posterior stroma were flatter. The reconstructions showed extensive projections from the corneal stromal cells, directed to adjacent cells and anteriorly towards the epithelium, but not penetrating through the basal lamina (black arrows). (**C**) To see the cell reconstructions in more detail and a clearer view of the cell extensions, please refer to Supplementary Video 3. Lens (L), Epithelium (E), Corneal stroma (S), Anterior stroma (AS), Posterior stroma (PS).

**Figure 6:**
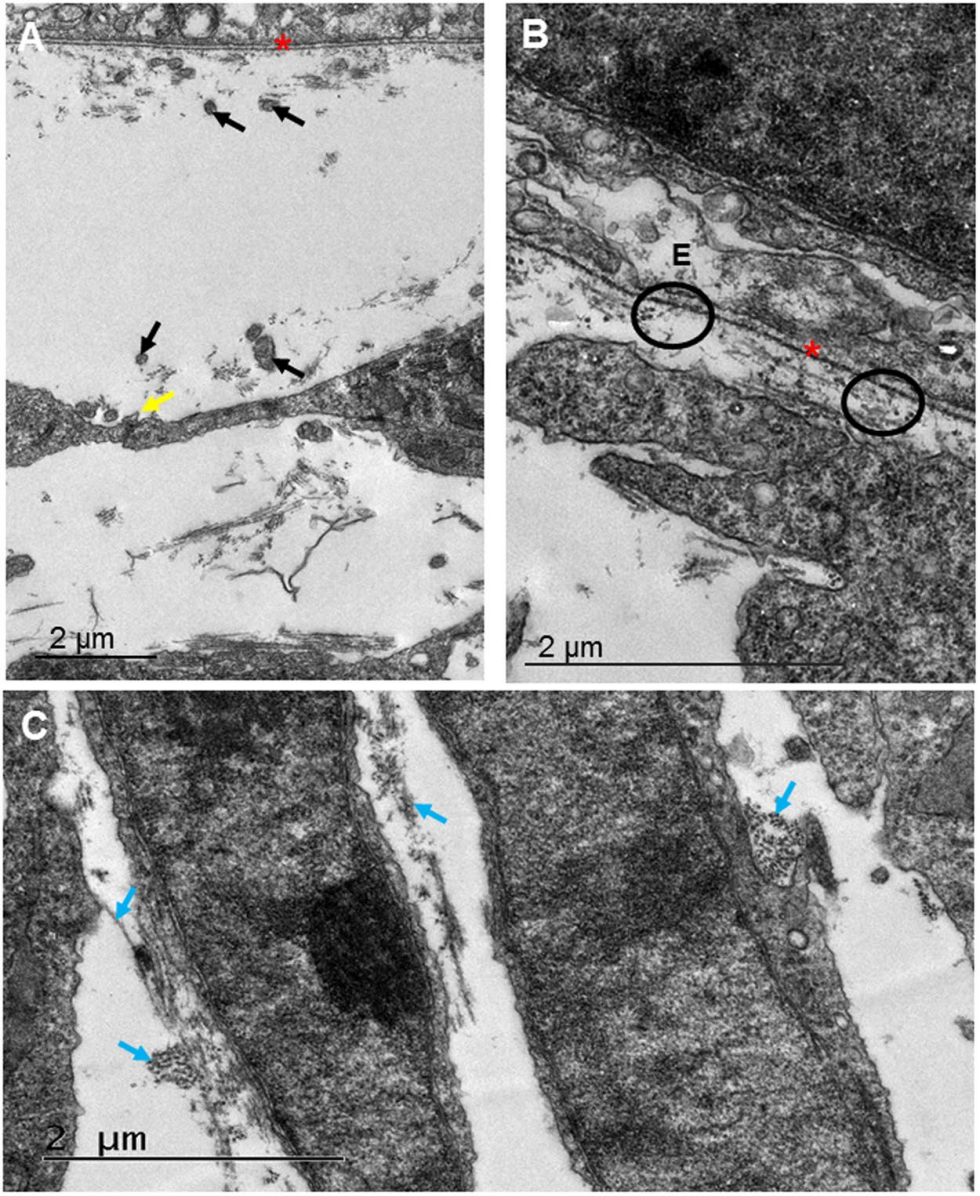
Transmission electron microscopy showed high resolution images of the developing cornea at E13. (**A**–**C**) Directly posterior to the basal lamina (red asterisk) collagen fibrils appeared to be closely associated with cell projections (black arrows). (**A**) The neighbouring cells projections were also seen to communicate (yellow arrow). (**A**) The collagen fibrils were randomly organised within the area posterior to the corneal epithelium (E), with some fibrils appearing to hang perpendicularly to neighbouring fibrils from the basal lamina (black circle). Within the central anterior cornea, the cells were larger than those in the posterior regions, and possessed a more stellate morphology. (**B**,**C**) Within the posterior cornea, the cells appeared flatter, with collagen associating around the cell membranes (blue arrows). (**C**) Small bundles of collagen fibrils were orthogonally orientated to one another (blue arrows). (**C**) Scale bar = 2 μm.

By E13, the cells displayed a stellate morphology within the anterior of the developing cornea. The cells within the posterior cornea possessed a flatter morphology, with adjacent cells in close proximity to one another (Fig. 5). These changes in cell morphology suggest that mesenchymal cells have differentiated into corneal stromal cells (Fig. 5). The 3-D reconstructions of the datasets showed an extensive communication system between the cells of the presumptive corneal stroma via cell projections. In addition to the cell projections which branched to neighbouring corneal stromal cells, extensions were seen to run anteriorly towards the basal lamina, appearing to communicate with the epithelium (Fig. 5 and supplementary video 3). The high resolution images taken by TEM showed small amounts of collagen fibril deposition, which was enhanced posterior to the basal lamina. An association between the collagen fibrils and corneal stromal cells was also seen (Fig. 5). High resolution imaging also identified cell projections making contact with adjacent cell projections; these projections extended throughout the corneal stroma and towards the basal lamina (Fig. 6). The collagen fibril bundles were orthogonally organised with respect to adjacent bundles, and were in close proximity to corneal stromal cell membranes (Fig. 6).

#### Stage E14

**Figure 7:**
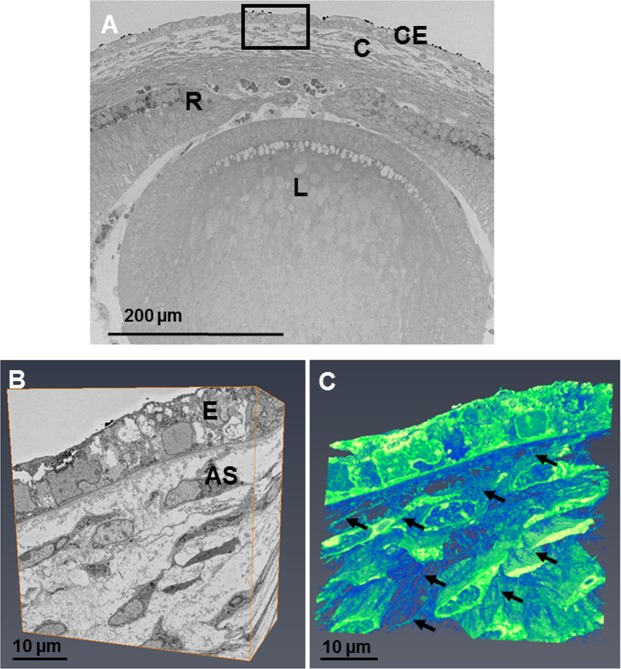
The developing eye at E14 was imaged at a low magnification (×360) showing the lens (L), retina (R), cornea (C) and corneal epithelium (E). (**A**) Reconstructions at a high magnification within the area of the black box were made following the method described in Fig. 1. (**A**,**B**) The reconstructions showed cells within the anterior stroma that appeared stellate, surrounded by extracellular space. Reconstructions showed extensive projections from the corneal stromal cells, directed towards adjacent cells and anteriorly towards the epithelium (**C**) (black arrows). To see the cell reconstructions in more detail and a clearer view of the cell extensions, please refer to Supplementary Video 4.

**Figure 8:**
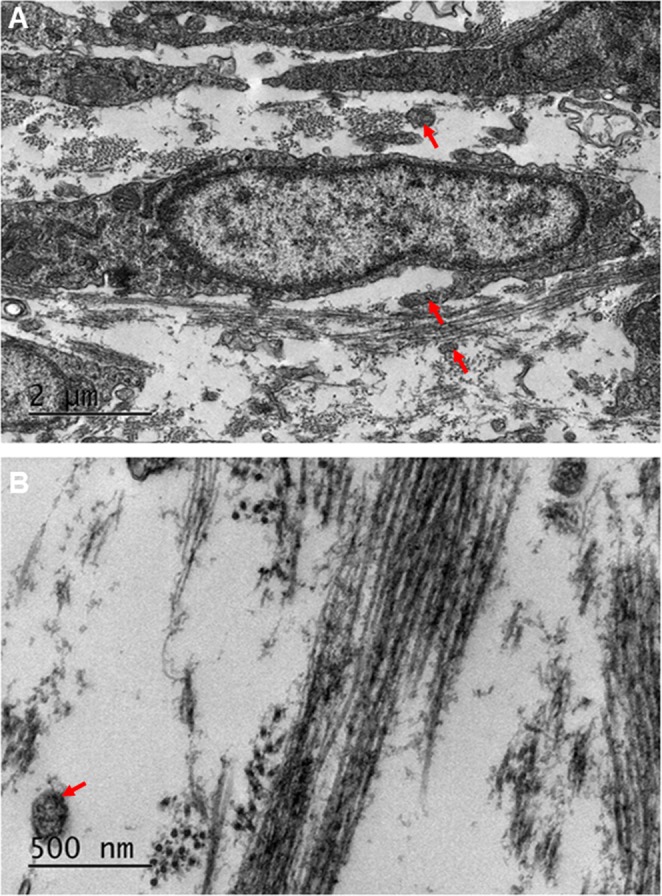
Transmission electron microscopy of the developing corneal stroma at E14 showed high resolution images of the collagen fibrils. (**A**,**B**) At lower magnification, the collagen fibrils were seen to be abundant, surrounding the cells of the corneal stroma. (**A**) The collagen fibrils appeared to have started to cluster within bundles, with the bundles arranged orthogonally between cells. (**A**) An abundance of cell projections was seen within the corneal stroma, with some associating with the collagen fibrils (**A**, red arrows). The collagen fibrils were imaged at a higher magnification to show the orthogonal arrangement more clearly (**B**).

Electron microscopy showed enhanced collagen fibril deposition and organisation with increased development (Figs 7 and 8). Three-dimensional reconstructions at E14 showed stellate cells within the anterior cornea, with cell projections communicating with adjacent cell projections (Fig. 7 and Supplementary Video 4). Corneal stromal cells in the posterior cornea displayed a lengthened and flat morphology. Analysis of the corneal stroma at E14 at a higher resolution using TEM showed collagen fibrils associating with cell membranes, appearing to form orthogonally organised collagen fibril bundles (Fig. 8). Cell projections were also seen to associate with the collagen fibrils, with many cell projections aligning in the direction in which collagen fibrils were organised (Fig. 8).

#### Stages E15 to E18

**Figure 9:**
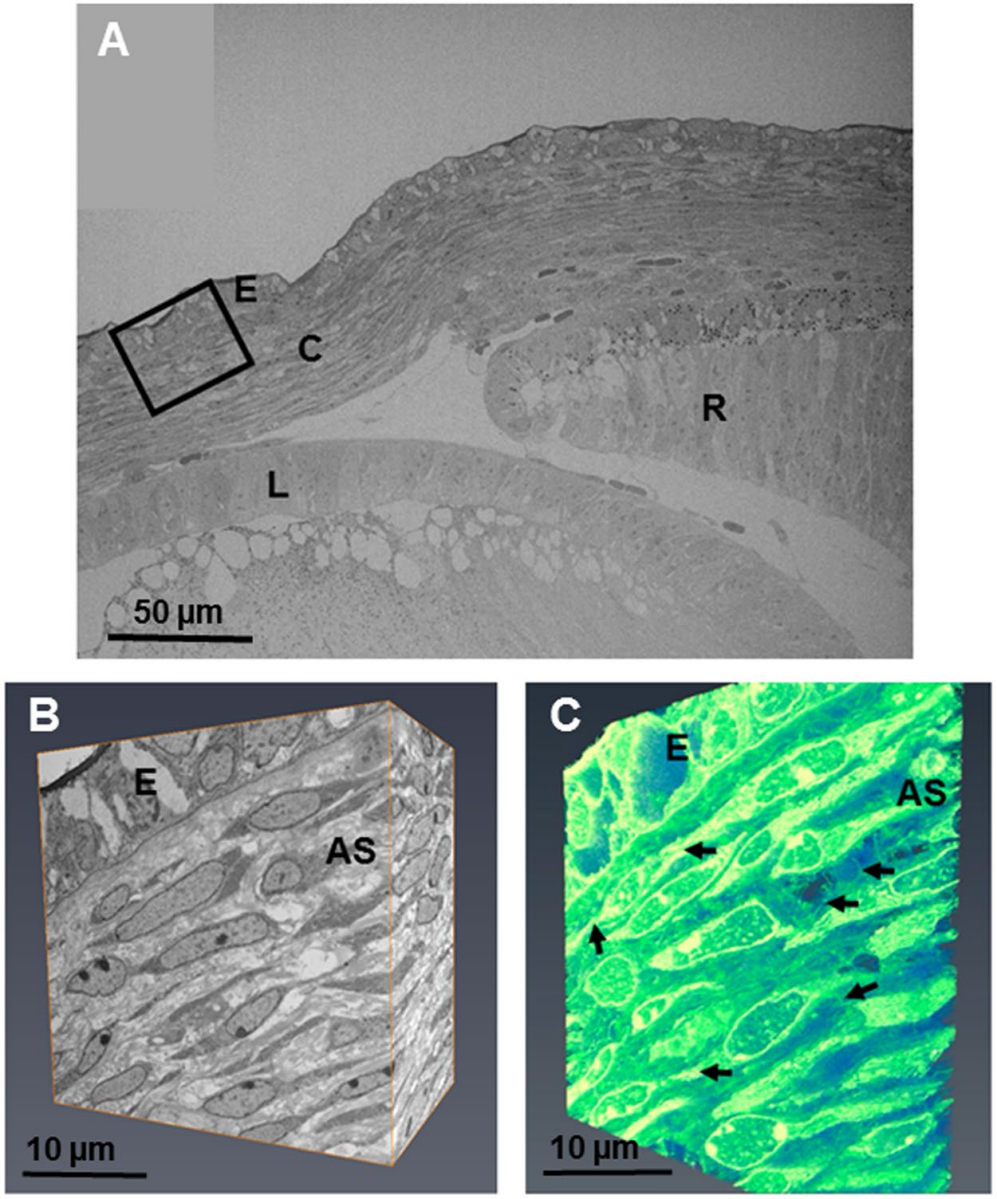
The developing eye at E15 was imaged at a low magnification (×540) and images stitched together to show the lens (L), retina (R), corneal stroma (C) and corneal epithelium (E). (**A**) Reconstructions at a high magnification within the area of the black box were made in the anterior corneal stroma (AS) following the method described in Fig. 1. (**A**,**B**) The reconstructions showed sub-epithelial cells to have flattened, now being similar to the cell morphology in the posterior cornea. The datasets were segmented using volren and showed extensive projections from the corneal stroma cells connecting to adjacent cells (Fig. **C**, black arrows). To see the reconstructions in more detail, please refer to Supplementary Video 5.

**Figure 10:**
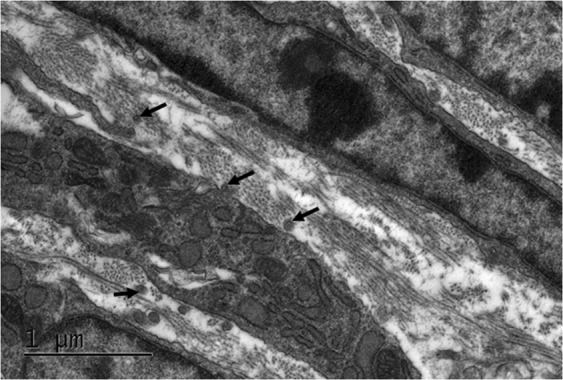
Transmission electron microscopy was used to obtain high magnification images of the developing corneal stroma at E15. Collagen bundles appeared to form into orthogonally organised lamellae between the corneal stromal cells. The direction of some collagen fibril bundles also appeared to align with the direction of alignment of some of the cellular extensions (black arrows).

**Figure 11:**
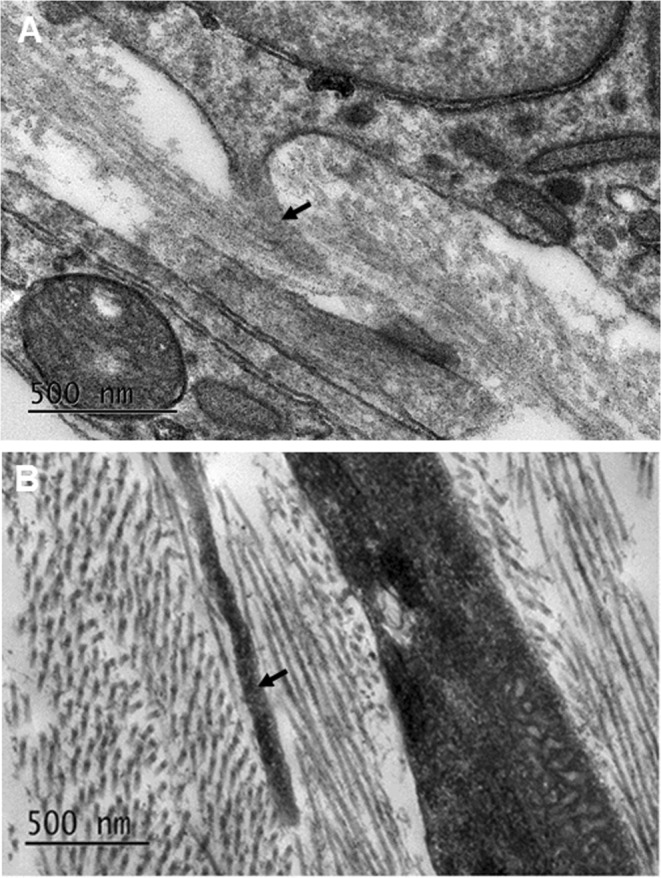
Transmission electron microscopy imaging of the developing corneal stromal cells at E16 (**A**) and E18 (**B**). With maturation, the amount of collagen fibrils was seen to increase until organised orthogonal lamellae were present. There was a tendency for cell projections (black arrow) within the corneal stroma of the developing corneas at E16 and E18 to align in the same direction as some of the immediately adjacent collagen fibrils.

Collagen fibril deposition had further increased by E15, with the cells orientated in a longitudinal arrangement (Fig. 9 and Video 5). At E15 the sub-epithelial cells appeared to have flattened, with a similar morphology to cells within the posterior cornea. High magnification imaging with TEM revealed bundles of collagen fibrils appearing to align within lamellae between adjacent parallel cells (Fig. 10). In addition, many of the cell processes appeared to align in the direction of collagen fibril organisation (Fig. 10). Through E16 to E18, the collagen fibrils continued to align with the direction of the majority of cellular projections (Fig. 11).

### Proteoglycan distributions

**Figure 12:**
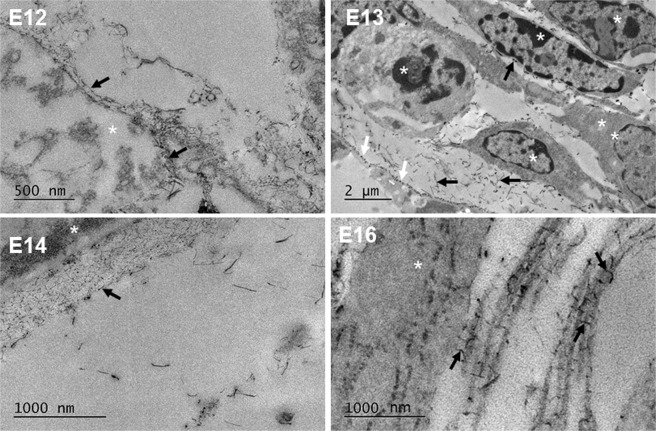
Cuprolinic blue positively stained the glycosaminoglycan (GAG) side chains of proteoglycans (PGs) with a dark contrast for transmission electron microscopy imaging. At E12, PGs (black arrows) initially associated with the surface of the mesenchymal cells (white asterisks). With increased development (E13) the PGs (black arrows) were found around cells (white asterisks) and independently within the extracellular matrix space. Some cell processes show regular attachment of PGs transverse to the direction of the process (white arrows). At E14, PGs (black arrow) were additionally found to accumulate posterior to the corneal epithelium (white asterisk), affilliated with the basal lamina. At E16, PGs were seen to lie between the cell (white asterisk) and collagen fibrils, and between adjacent collagen fibrils (black arrows).

Cuprolinic blue positively stained the glycosaminoglycan (GAG) side chains of proteoglycans (PGs) between E12-E18 of prenatal mouse development. From E12 of mouse development, PGs were seen to be membrane bound, associating around the mesenchymal cells (Fig. 12). As development progressed from E12 to E18, PGs were located close to corneal stromal cells. At E13, they appeared to attach in a regular manner along the cell processes, as well as in the intercellular matrix. PGs were shown to accumulate posterior to the developing corneal epithelium at E14, and also appeared within the developing corneal stroma. Collagen fibrils increased in size from E16 and the PGs were found to associate intimately with adjacent collagen fibrils in a manner reminiscent of the adult stroma, as well as between the collagen fibrils and the corneal stromal cells (Fig. 12).

### Immunofluorescence

**Figure 13:**
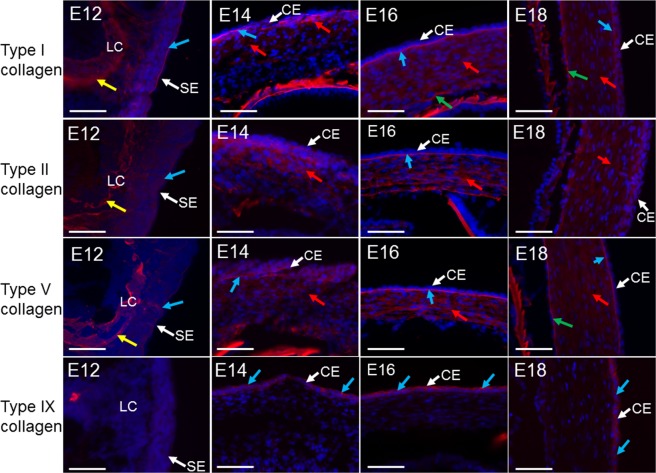
Collagen distributions within the developing mouse cornea. All corneal epithelia are at the top in E14 and E16, and to the right in E12 and E18. Type I collagen was initially seen within the surface ectoderm (SE) (blue arrow) and lens capsule (LC) (yellow arrow) at E12, with enhanced expression posterior to the corneal epithelium (CE) at E14 (blue arrows) and within the anterior corneal stroma (red arrows). Expression was shown throughout the corneal stroma from E16-E18 (red arrows). Type I collagen was enhanced posteriorly to the corneal epithelium at E16 (blue arrows) as well as anterior to the endothelium at E16 and E18 (green arrows). Type II collagen was initially seen within the surface ectoderm (blue arrow) and lens capsule (yellow arrow) at E12, enhancing within the anterior cornea from E14 and throughout the corneal stroma between E16-E18 (red arrow). Expression was also increased posterior to the corneal epithelium at E16 (blue arrow). Type V collagen was expressed within the surface ectoderm as a localised line (blue arrow) as well as within the lens capsule (yellow arrow) from E12, type V collagen expressed posteriorly to the corneal epithelium from E14 (blue arrow) and throughout the corneal stroma from E16-E18 (red arrows). An enhanced expression was also seen posteriorly to the corneal epithelium from E16-E18 (blue arrow) and anteriorly to the endothelium from E18 (green arrow). Type IX collagen was not seen at E12, with a little expression associated with the corneal epithelium at E14 (blue arrows). Expression enhanced between E16-E18 within the corneal epithelium (blue arrows). No type IX collagen was seen within other areas of the cornea during development. No expression was apparent within the no primary antibody control and the rabbit IgG control images (data not shown). Adult corneas were used as a positive control for all antibodies (data not shown). Blue (DAPI – cell nucleus), red (collagen). Scale bar = 50 μm.

Types I, II, V and IX collagen were analysed throughout corneal development from E12-E18 (Fig. 13). Types I, II and V collagen were found to be stained as a localised line along the surface ectoderm as well as within the lens capsule at E12. All collagens were found to increase with development. Collagen types I, II and V were  confined to the anterior corneal stroma in E14, but were found throughout the thickness of the corneal stroma by E16. The prominent collagen type I staining at E16 and E18 appeared to be closely associated with the epithelial basement membrane from E12, whereas a patchy and more prominent expression was seen at E16 and E18 respectively. The expression appeared to be related to the maturation of the corneal endothelium and also appeared to be closely associated with Descemet’s membrane. Type IX collagen was initially expressed within the corneal epithelium from E14, continuing to be expressed in the corneal epithelium between E16-E18. No expression of type IX collagen was found within the corneal stroma. All controls, including a no primary antibody and a rabbit IgG control for each antibody, showed no positive staining (data not shown).

**Figure 14:**
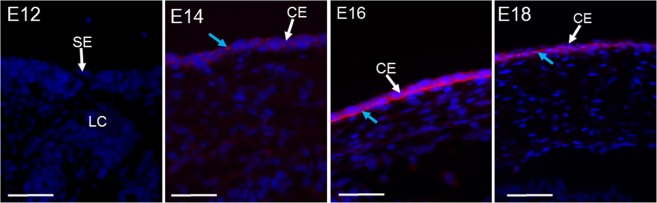
Type VII collagen expression in corneal development. Expression initially appeared in the corneal epithelium (CE) of E14 (blue arrow), with no expression associating with the surface ectoderm (SE) at E12. By E16 type VII collagen (blue arrow) appeared localised along the epithelium basement membrane. The positive control of an adult mouse cornea (data not presented) showed the usual type VII collagen expression along the basement membrane of the corneal epithelium. No expression was present in the no primary antibody control and the rabbit IgG control images (data not shown). Blue (DAPI – cell nucleus), red (type VII collagen). Scale bars = 50 μm.

To confirm the electron microscopic evidence of anchoring filaments within the developing mouse cornea, type VII collagen expression was labelled (Fig. 14). The immunofluorescence results showed that type VII collagen was associated with the corneal epithelium from E14, increasing with corneal development to localise along the basement layer of the corneal epithelium.

## Discussion

Utilising three-dimensional electron microscopy techniques to image the structural development of the prenatal mouse cornea from E10-E18, this study identified extensive cell extensions within the developing mammalian corneal stroma that spanned adjacent cells and showed a strong tendency to align with the direction of collagen fibrils. In addition, unlike the well-studied avian cornea, no acellular primary stroma was required to initiate a neural crest cell migration into the presumptive corneal stroma, confirming previous studies^13^. This paper demonstrated the presence of types I, II and V collagen posterior to the surface ectoderm from E12 which spread across the developing corneal stroma by E14, initially being synthesised in the anterior stroma. Type IX collagen was observed at the later stage of E14 within the corneal epithelium. Our results provide structural information about the prenatal mouse cornea, which can be utilised to further understand mammalian development, whilst providing a comparator for the avian developmental model.

The developing mouse cornea between E10-E12 showed the surface ectoderm overlying the lens with migrating neural crest cells infiltrating the presumptive cornea. There was no evidence of an acellular collagen matrix layer along which the cells were migrating, unlike the primary stroma in the well-reviewed avian cornea^4^. In addition, the cells within the corneal stroma between E10-E12 appeared to be responsible for synthesising collagen, with cells containing secretory organelles. Our results showed an increased density of mesenchymal cells from E10 to E12, with an enhanced extracellular matrix space around the cells from E13. This dense packing of mesenchymal cells between E10-E12 with a greater extracellular matrix space at later developmental stages has previously been described in the mouse cornea^19^. Interestingly, unorganised strands of material were seen to extend extracellularly from the surface ectoderm at E10 of mouse development; these strands could be precursor collagen fibrils or other extracellular matrix components. Within avian corneal development, the secretion of an acellular primary stroma from the presumptive corneal epithelium and endothelium is crucial to stimulate the second migration of cells to synthesise the mature corneal stroma^20^. The mouse cornea is known to have only one mesenchymal cell migration, whose cells develop the corneal stroma and later the corneal endothelium^21^. In addition, type IX collagen was seen within the developing avian corneal stroma, whose role is to maintain the primary stroma and stimulate its breakdown once the cornea has swollen and cells have migrated in^7^. Type IX collagen was not expressed within the mouse cornea at these early stages, suggesting that its role in maintaining a primary stroma is not required. These results confirm previous studies of a collagenous primary stroma not being synthesised within the developing mouse cornea and that it is not required for the subsequent cell migration, where the incoming cells lay down the mature corneal stroma^21^.

At E13, the cells’ morphology became more stellate, suggesting that they had differentiated from mesenchymal cells to corneal stromal cells. Collagen fibril deposition within the electron microscopy studies was initially seen at E13, increasing in quantity and alignment as development progressed. A striking feature of the SBF-SEM 3-D reconstructions was a highly extensive interactive system between the cells of the corneal stroma. The cell processes of the presumptive corneal stroma extended to adjacent corneal stromal cell projections, as well as directing anteriorly towards the basal lamina, suggesting a possible communication network between the stromal cells, and between the stromal cells and the developing epithelium. The cell extensions within the three-dimensional models were seen to communicate with those from adjacent corneal stromal cells at all ages analysed between E13-E18. The association between cell projections of adjacent corneal stromal cells has been previously described as being connected through gap junctions for communications^22^. Cell communications have also been seen in the rabbit cornea between the mesenchymal cells and the corneal epithelium, and these communications are thought to maintain a stem cell niche for regeneration^23^. However, the corneal stromal cell projections which branched anteriorly towards the epithelium showed no cell-cell contacts, suggesting that, if they are communicating, it is most probably through chemical signalling or mechanotransduction. This extensive association of the cell projections leads to the suggestion that direct communication between the cells is important during these stages of corneal development, and any disruption to this system could affect the physiological development of the cornea.

TEM imaging of the developing mouse cornea yielded high resolution images which revealed intricate details of the corneal stroma that were not seen in the SBF-SEM data-sets. The TEM images appeared to indicate collagen fibrils initially secreted from the presumptive corneal stromal cells. The TEM images showed an enhancement of collagen fibrils posterior to the basal lamina of the embryonic corneal epithelium from E13. Some of these fibrils appeared to organise perpendicularly to neighbouring fibrils. We hypothesise that these fibrils are anchoring filaments from their position within the developing cornea. To test this hypothesis, type VII collagen was found to express posteriorly to the corneal epithelium, in close proximity to the basal lamina. Even though this does not specifically identify the filaments seen in the TEM image, the positive expression of type VII collagen supports our suggestion that the structures are anchoring filaments. To our knowledge, this is the first study to propose anchoring filaments within the developing mouse cornea. Previous studies have identified the filaments posterior to the basal lamina within the rabbit and human developing corneas, thus showing similarities between developing mammalian models^24^.

An interesting observation throughout corneal development was the potential association of the collagen fibrils with the cell membranes and cell extensions, which has been previously seen in the avian cornea^16^. From the initial sign of collagen fibril deposition, bundles of collagen fibrils associated around cell membranes. This close association of collagen fibrils around cell membranes was shown in previous mouse corneal studies^13^. In addition, collagen fibril assembly and deposition is associated with cell membranes in tendon development^17^. During tendon development, collagen is packaged within plasma membranes termed fibripositors, which extrude collagen fibrils out of the cell, in the same direction as the fibripositors parallel alignment^17,25^. In the mouse cornea, we found no evidence of fibripositors directing collagen fibril alignment. It is known that collagen molecules, in the absence of cells, contain information to assemble, but do not have the required information to align. This suggested that cells provide the necessary alignment queues^26^. It appears that collagen alignment in the cornea proceeds via a different mechanism to what has been described in tendon development, with collagen being secreted out of the stromal cells in the absence of fibripositors, followed later by the cell projections aligning the collagen fibrils. This method of alignment was similarly described in the avian cornea, with collagen fibrils associated with cell membranes^16^. However, the cell surface compartments containing collagen identified in these previous studies were not seen in the mouse cornea^16^.

The collagen fibril bundles at E15 appeared to pack within orthogonal lamellae between cells, with the cell extensions aligned with the direction of collagen fibrils in the corneal stroma. In addition, the combined observations that cell alignment occurs prior to collagen deposition, more collagen fibrils are aligned in the posterior cornea where more of the cells are aligned than in the anterior cornea at E14, and cellular processes are aligned with the fibrils at later time points, supports our hypothesis that cell extensions could be responsible for organising collagen fibrils. Within the avian species, collagen fibril bundles have also been shown to closely associate with the cell membranes of keratocytes^16^. Cell extensions termed keratopodia within avian development have been shown to orientate and organise collagen into lamellae, with cell rotation occurring before collagen fibril organisation^18,27^. The results in the present study support previous findings of cell extension alignment with collagen fibrils. However, Koudouna *et al*., 2018 highlighted the importance of understanding the mechanisms that organise collagen fibrils during corneal development and further research should focus on elucidating these mechanisms.

Types I, II and V collagen expressed as a localised line associated with the developing surface ectoderm at E12. Similar expression is seen in the avian species where types I and II collagens are initially laid down by the corneal epithelium^5^. However, no evidence in the mouse cornea has shown the synthesis of a collagenous primary stroma. Even though an acellular organised collagen matrix was absent in the mouse cornea, extracellular matrix material was seen from E10 of mouse development and could represent precursor collagen fibrils; but specific identification of these fibrils is needed. Type I, II and V collagen strongly expressed in the anterior aspect of the corneal stroma at E14, before appearing throughout the corneal stroma from E16-E18. The staining profiles of collagen were enhanced within the anterior aspect of the developing cornea and the cell morphology appeared rounder in the anterior cornea with a greater amount of extracellular space surrounding the cells. This implies that the cells within the anterior corneal stroma are responsible for synthesising and depositing collagen within the corneal stroma. The assembly of type I collagen is crucial to provide the cornea with structural support and transparency. Type I collagen knockout studies in the mouse have shown collagen fibrils from E16 to be reduced in thickness in comparison to the wild type cornea, disrupting the corneal stroma’s structure^28^. These results suggest that from an early stage in mouse corneal development the structure of the corneal stroma is already being established and relies on the assembly of type I collagen.

Type IX collagen did not show a similar distribution of expression to that described in the avian cornea. Type IX collagen was not seen in the initial stages of corneal development, first appearing associated with the corneal epithelium at E14 and continuing to be expressed to E18. The enhancement of type IX collagen correlated with an increase in type II collagen. Even though the expression profiles appeared different between types IX and II collagen, the enhanced labelling associated with the corneal epithelium could indicate an interaction of type IX collagen with the fibrils of type II collagen at this interface. Type IX collagen expression is usually found in tissues rich in type II collagen, often associating together for stabilisation of the fibrils^29–31^. Type IX collagen expression has also been previously identified within the corneal epithelium of the developing human foetal cornea, similar to the mouse development results shown in the present study^32^. These results indicate that type IX collagen plays a different role within the corneal epithelium in mammalian development to that seen in the avian cornea. Type IX collagen is thought to maintain the primary stroma in avian development, with MMP-2 and MT3-MMP initiating its breakdown for mesenchymal cell migration^8^. These events not occurring in mouse corneal development may be one reason for the lack of type IX collagen expression within the mouse corneal stroma.

Proteoglycans (PGs) were shown to have a close association between the developing corneal stromal cells, basal lamina and collagen fibrils. PGs were identified before the deposition of collagen fibrils, accumulating posteriorly to the corneal epithelium. Within lung tissue, PGs are seen to accumulate posterior to the epithelium to regulate epithelial cell function^33^. The PGs posterior to the mouse corneal epithelium suggests a role in regulating the function and development of the epithelial cells. Previous studies have suggested PGs regulate mesenchymal cell migration and their differentiation into keratocytes^34^. PGs also have a fundamental role in maintaining collagen fibril alignment in the mature cornea. The presence of PGs in prenatal development indicates that this role initiates at an earlier stage than previously thought. The observation of PGs linking the corneal stromal cells to the collagen fibrils could suggest that the PGs play a role in the initial alignment of the collagen fibrils. PGs are well known to play a role in maintaining a transparent cornea, with knockout studies showing the fusion of adjacent collagen fibrils and increased opacity of the cornea^35–37^. Specifically, lumican has previously been shown to prevent lateral fibril growth of newly deposited collagen fibril intermediates in mouse neonatal development, supporting the role of PGs regulating the diameter of collagen fibrils^38^. Within avian development, the increase in sulphation of GAGs on keratan sulphate PGs is thought to achieve matrix hydration and transparency^39^. The adult mouse cornea has reduced sulphated keratan sulphate PGs, having shorter GAG chains with increased N-acetyl lactosamine disaccharides lacking extended domains with disulphated disaccharides^40^. This reduction of sulphated PGs may also be seen in the prenatal mouse cornea compared to the developing avian cornea. These results demonstrate that PGs play a role in developing a transparent cornea. Future studies should focus on identifying the specific type of PGs at each developmental stage of mouse development.

It should also be noted that other extracellular matrix proteins are crucial for successful development of the cornea. Fibronectin and hyaluronic acid have previously been shown to act as a substrate for migrating cells^41,42^. Within avian corneal development, the migrating cells that construct the cornea are seen to associate with a fibronectin substrate^43^. These extracellular components are likely to be present in early mouse corneal development, but their localisation should be further explored to confirm this hypothesis^41,42^. Similarly, for successful cell migration and morphogenesis during development, basement membranes may play an important role^44^, so further exploration of the components embedded in basement membranes, such as laminins and cell receptors, could reveal crucial information concerning the mechanisms that direct corneal assembly^45^.

In summary, this paper has used 3-D imaging techniques to study the pre-natal development of the mouse cornea, focusing on the initial synthesis and alignment of collagen fibrils. The 3-D imaging technique highlights novel findings of extensive cell projections between the developing cells of the corneal stroma and the epithelium. The apparent alignment of cell projections with collagen fibril orientation in the mouse corneal stroma suggests a role for the cells in the organisation of the collagen fibrils, however further work is needed to support this hypothesis. Immunofluorescence experiments in this study have demonstrated that types I, II and V collagen are mainly expressed within the developing corneal mouse stroma and type IX is associated with the corneal epithelium. It was also evident that a primary stroma is not present within the developing mouse cornea, with no organised acellular matrix seen with neural crest cell migration. In conclusion, these results show a clear divergence between the development of the avian and mouse cornea, but a similarity with collagen fibril organisation via associations with cell projections. In addition, comparison of the mouse cornea to other developmental mammalian models, including the human and rabbit, shows some similarities. Future studies should focus on understanding the alignment of collagen fibrils within the mammalian corneal matrix in developing a transparent cornea, elucidating the molecular and biomechanical cues that organise the collagen fibrils.

## Methods

### Tissue collection

All embryonic mouse tissue was retrieved from time-mated pregnant mice (C57BL/6) following cervical dislocation at a Schedule 1-approved designated establishment in accordance with Animals (scientific procedure) Act 1986 (United Kingdom) and Home Office (United Kingdom) guidance rules. The experimental protocol was approved by Cardiff University ethics committee. For each age between E10-E18, 6 un-paired whole eyes were analysed.

### Deerinck staining method

A modified Deerinck method was used to enhance cellular components for electron microscopy analysis^46^. Eyes were fixed in Karnovsky’s fixative for 3 hrs at 4 °C^47^. After fixation samples were washed in cacodylate buffer for 10 mins before being transferred into a solution containing 1.5% potassium ferricyanide/1% osmium tetroxide in cacodylate buffer for 1 hr. After washing in distilled water for 30 mins, samples were then placed sequentially in 1% aqueous thiocarbohydazide, 1% osmium tetroxide and 1% aqueous uranyl acetate, each for 1 hr. All the staining steps were followed by 30 mins distilled water washing steps.

Samples were then incubated in an oven for 1 hr in a solution of lead aspartate at 60 °C. After lead staining, the samples were washed in two changes of distilled water for 30 mins. Finally, the samples were dehydrated in an ethanol series from 70% through to 100% and via acetone infiltrated and embedded in CY212 (TAAB) epoxy resin and polymerised for 48 hr at 60 °C.

### Cuprolinic blue method

Cuprolinic blue staining was employed to stain the glycosaminoglycan (GAG) side chains of proteoglycans^48^. Positive staining is achieved due to the cationic properties of the stain initiating a metachromatic reaction with the anionic GAG chains. Fresh samples were immersed in vials for 24 hrs containing 2.5% glutaraldehyde in 25 mM sodium acetate buffer, pH 5.7 containing 0.1 M magnesium chloride and 0.05% cuprolinic blue (Merelex Corporation). The samples were rinsed in sodium acetate buffer and washed over 10 mins in aqueous 0.5% sodium tungstate. Finally, the samples were dehydrated in an ethanol series from 70% through to 100% and via acetone infiltrated and embedded in CY212 (TAAB) epoxy resin and polymerised for 48 hr at 60 °C.

### Serial-block face scanning electron microscopy (SBF-SEM)

Processed Deerinck-stained samples were mounted onto a Gatan specimen pin and coated with silver conductive epoxy adhesive (TAAB laboratories). The pin was sputtered with 8 nm of gold using the Leica ACE 200 and placed inside the Zeiss Sigma VP FEG SEM equipped with a Gatan 3View system. Automated serial sectioning was undertaken of the block face surface every 50 nm until a dataset of 1000 images was acquired. 3-D reconstructions of the datasets using the automated volren segmentation function were composed using Amira 6.4 software (FEI, Mérignac, France).

### Transmission electron microscopy

#### Deerinck stained samples

Ultrathin sections were cut (90 nm) using the Leica UC6 ultra-microtome, collected on 300 hexagonal copper grids and analysed using the JEOL 1010 transmission electron microscope.

#### Cuprolinic blue stained samples

Ultrathin sections (90 nm) were cut on the Leica UC6 ultra-microtome and collected on 300 hexagonal copper grids. The copper grids were further stained with 0.5% aqueous uranyl acetate for 10 mins. Once stained, grids were washed with distilled water and left to dry for 24 hrs before being imaged with the JEOL 1010 transmission electron microscope.

### Immunohistochemistry

Fresh samples were frozen over dry ice in optical cutting temperature compound (OCT) and cryosectioned transversely (5 μm) using a Leica CM3050 S cryostat, collecting sections on Superfrost Plus Slides (Thermo Scientific, UK). The cryosections were circumscribed with a water repellent delimiting pen (ImmEdge Hydrophobic Barrier PAP pen, Vector labs) before being rehydrated with phosphate buffered saline solution (PBST [Tween-20, 0.1% Tween-20, 0.05 M, pH 7.3]). Cryosections were blocked with 5% horse serum in PBST for 20 mins^49^. Primary antibodies were added to the cryosections and incubated for 24 hrs at 4 °C, washed in PBST (3 changes over 10 mins) before adding secondary antibodies. Cryosections were incubated for 5 hrs at room temperature before secondary antibodies were washed off with PBST. Cover slips (VWR International) were added to the cryosections using VECTASHIELD HardSet Antifade Mounting Medium, containing DAPI to label nuclei blue. The first negative controls included sections with no primary antibodies and the sections included immunoglobulin controls substituted with the primary antibody application. Cryosections were imaged using the Olympus BX61 epifluorescence microscope, equipped with an F-view Digital camera using ×10, ×20 and ×40 objectives.

### Antibodies

Rabbit Polyclonal (Type I collagen, type II collagen, type V collagen, type IX collagen and type VII collagen) primary antibodies were used^50–53^. Dylight 594 Horse Anti-Rabbit (vector labs) was applied as the secondary antibody at 1:200 dilution.

## Supplementary information

Supplementary material

Supplementary video 1

Supplementary video 2

Supplementary video 3

Supplementary video 4

Supplementary video 5

## Data Availability

The analysed datasets from this study are available from the corresponding author upon request.
