# Supplementary material for: Three-dimensional imaging of the extracellular matrix and cell interactions in the developing prenatal mouse cornea
Source: Sci Rep. 2019 Aug 2;9:11277. doi: 10.1038/s41598-019-47653-z (PMC6677755; doi:10.1038/s41598-019-47653-z)
Supplement: Supplementary file 1 — Supplementary material [file 41598_2019_47653_MOESM1_ESM.pdf]

Three-dimensional imaging of the extracellular matrix and cell interactions in the developing prenatal mouse cornea

Eleanor M. Feneck, Philip N. Lewis, Keith M. Meek

Structural Biophysics Research Group, School of Optometry and Vision Sciences, Cardiff University, Maindy Road, Cardiff, CF24 4HQ, UK

Corresponding author; Professor Keith Meek – meekkm@cardiff.ac.uk

Supplementary videos

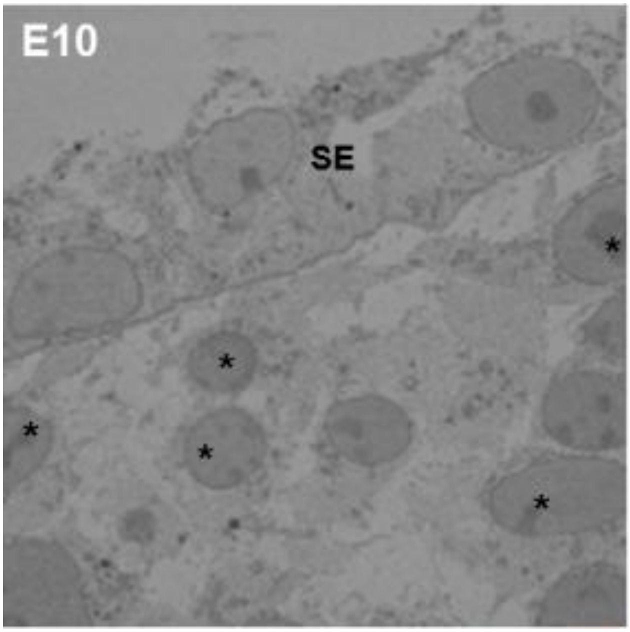

Supplementary Video 1. A video of the 3-D reconstructions of the E10 SBF-SEM dataset between the surface ectoderm (SE) and migrating neural crest cells (black asterisks).

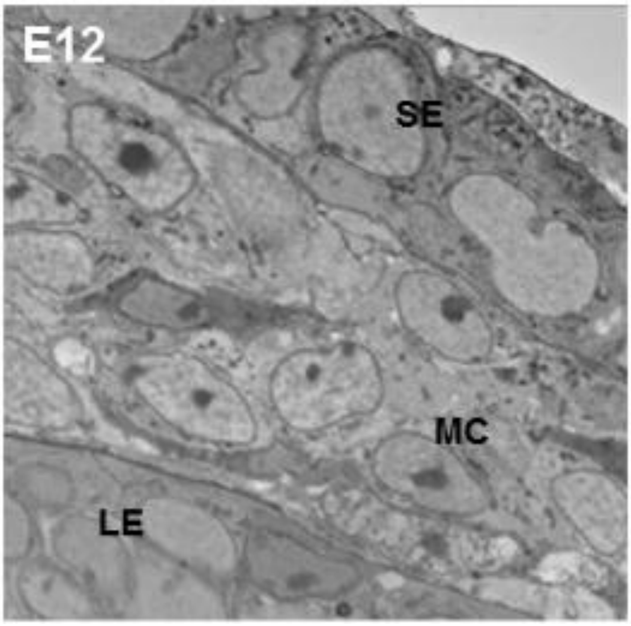

Supplementary Video 2. A video of the 3-D reconstructions of the E12 SBF-SEM dataset of the mesenchymal cells (MC) between the surface ectoderm (SE) and lens epithelium (LE).

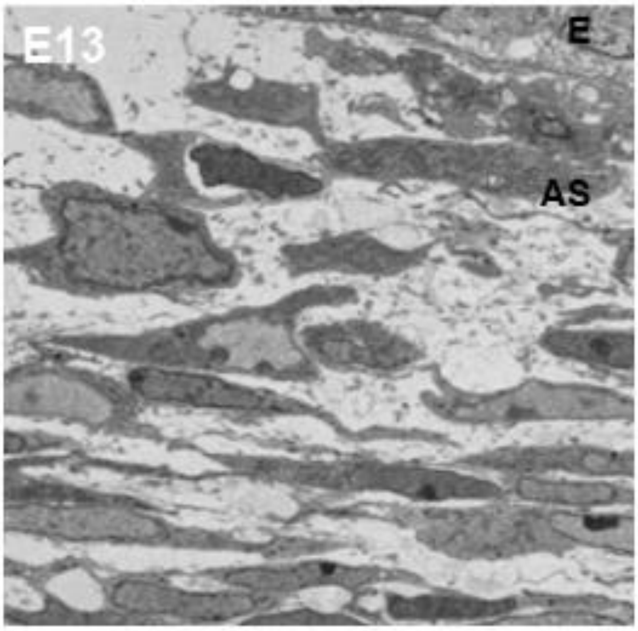

Supplementary Video 3. A video of the 3-D reconstructions of the E13 SBF-SEM dataset within the anterior corneal stroma (AS). Epithelium (E).

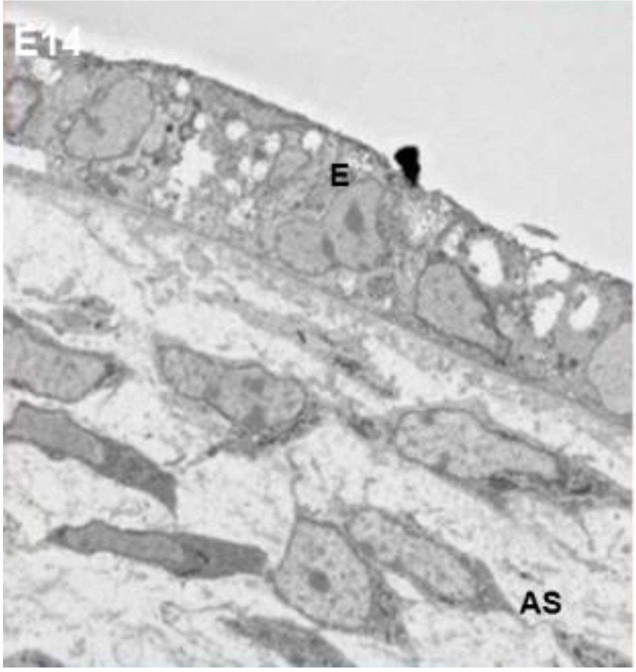

Supplementary video 4. A video of the 3-D reconstructions from the E14 SBF-SEM dataset of the corneal epithelium (E) and anterior corneal stroma (AS).

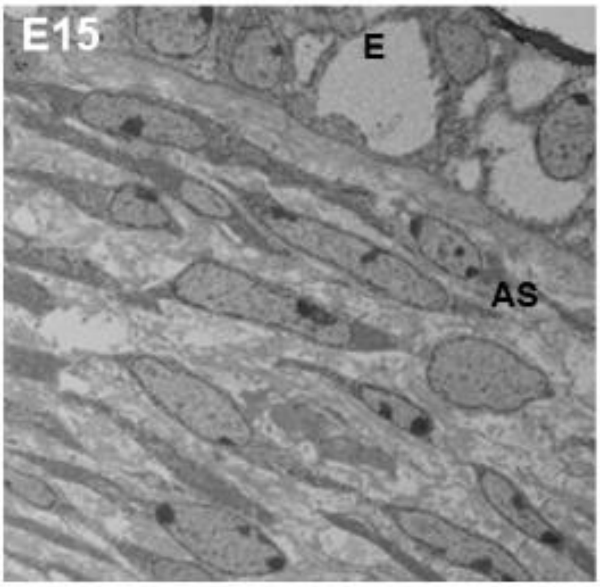

Supplementary video 5. A video of the 3-D reconstructions from the E15 SBF-SEM dataset of the corneal epithelium (CE) and anterior stroma (AS).
